# Two hands are better than one: Perceptual benefits by bimanual movements

**DOI:** 10.1167/jov.20.10.16

**Published:** 2020-10-15

**Authors:** Marnix Naber, Joris Elshout, Stefan Van der Stigchel

**Affiliations:** Experimental Psychology, Helmholtz Institute, Utrecht University, The Netherlands; Experimental Psychology, Helmholtz Institute, Utrecht University, The Netherlands; Experimental Psychology, Helmholtz Institute, Utrecht University, The Netherlands

**Keywords:** perceptual benefits, attention, motor control, motoric aiming, hand movements

## Abstract

Before looking at or reaching for an object, the focus of attention is first allocated to the movement object. Here we investigated whether the strength of these pre-motor shifts of attention cumulates if an object is targeted by multiple effectors (eyes and hands). A total of 29 participants were tested on a visuomotor task. They were cued to move gaze, the left hand, right hand, or both (one to three effectors) to a common object or to different peripheral objects. Before the movements, eight possible objects briefly changed form, of which one was a distinct probe. Results showed that the average recognition of the probe's identity change increased as more effectors targeted this object. For example, performance was higher when two hands as compared to one hand were moved to the probe. This effect remained evident despite the detrimental effect on performance of the increase in motor task complexity of moving two hands as compared to one hand. The accumulation of recognition improvements as a function of the number of effectors that successfully target the probe points at parallel and presumably independent mechanisms for hand- and eye-coordination that evoke pre-motor shifts of attention.

## Introduction

Whenever a person reaches or saccades to an object in the world, this is generally because the object attracted the focus of attention and/or there is an intention to interact with this object. It therefore comes as no surprise, perhaps, that visual attention and the preparation of motor movements are tightly linked. Rizzolatti, Riggio, Dscola and Umiltá (1987) first formulated the premotor theory of attention which postulates that attention is directed to a movement end location when a goal directed motor action is planned, irrespective of whether that motor movement is executed or not (Sheliga, Riggio, & Rizzolatti, 1994). These premotor shifts of attention are important to ensure motor accuracy and to integrate information while making movements across a scene (Zhao, Gersch, Schnitzer, Dosher, & Kowler, 2012). By allocating the focus of attention to an upcoming target location, visual information processing of the movement target is enhanced, with benefits for both transsaccadic perception and action as a result (Deubel & Schneider, 1996; Kowler, Anderson, Dosher, & Blaser, 1995). Due to the presaccadic shift of attention, the presaccadically-acquired information can be temporarily stored in visual working memory, allowing for quick transsaccadic integration after the saccade (Van der Stigchel & Hollingworth, 2018). Although these premotor shifts of attention have predominantly been investigated for saccadic eye movements (Deubel & Schneider, 1996; Montagnini & Castet, 2007; Baldauf & Deubel, 2008a), fewer studies have shown that the same principle holds for planning a reach, pointing or grapping movement (Deubel, Schneider, & Paprotta, 1998; Craighero, Fadiga, Rizzolatti, & Umiltà, 1999; Baldauf & Deubel, 2008b; Hesse & Deubel, 2011; Gilster, Hesse, & Deubel, 2012). In these experiments, the effect of a hand movement on perception is tested while the eyes remain fixated at a central fixation cross. Discrimination performance is higher for a probe targeted by the hand movement as compared to an untargeted probe.

In daily life, hand and eye (“effectors”) movements often co-occur (Neggers & Bekkering, 2000). When participants prepare coordinated eye and hand movements, attention is allocated in parallel to the targets of both the eye and the hand movement. When eye and hand movements are executed to a common target, this results in an additional boost of attention toward that location, compared to the effects of a single movement (Hanning, Aagten-Murphy, & Deubel, 2018; Jonikaitis & Deubel, 2011). The attentional allocation for different effectors has been claimed to be independent, meaning that there is a separate attentional resource for eye and hand movements. Interestingly, however, this boost of attention could not be replicated in a study by Khan, Song, and McPeek (2011), who found that combined eye-hand movements to a common target did not improve performance on a shape discrimination task compared to either an eye or hand movement alone (Khan et al., 2011). Moreover, they concluded that the eye movements dominate over hand movements if executed simultaneously. However, the number of participants in this study was relatively low (n = 5), with one of these participants actually showing a boost of attention for combined eye-hand movements.

Until now, no studies have focused on premovement shifts of attention during *bimanual* pointing movements to a common target and its interaction with the presaccadic shift of attention. Humans can accurately execute movements with both hands simultaneously to different targets, while gaze is at another spatial location (e.g., juggling, martial arts, typing on a keyboard). It is suggested that spatial attention can be distributed in parallel during bimanual hand movements when the eyes maintain fixation (Baldauf & Deubel, 2008b). This raises the question whether coordinated bimanual hand movements to common targets can potentially boost attention even further and how these bimanual hand movements interact with additional eye movements.

The goal of the present experiment was threefold: (1) replicate the findings of Deubel and colleagues, (2) extend these findings by adding a third effector (eye, left hand and right hand), and (3) study the effect of motor task complexity (i.e., variations in task difficulty related to the number of effectors moving) on the pre-movement shifts of attention. More specifically, we will examine whether both hands have separate resources to direct attention and whether adding a third effector (eyes) leads to worse perception, because of increased motor task complexity, or better perception because of the additional allocation of attentional resources. Participants were centrally cued to move either gaze or a single hand (one effector), both gaze and a hand or two hands (two effectors), or gaze and two hands (three effectors) to a common object (effector congruent) or multiple, separate objects (effector incongruent). The objects suddenly and briefly changed identity before the actual initiation of the movements. Participants needed to identify one distinct object (the probe) and report its identity (E or 3) through a delayed response (two-alternative-forced choice task) after the movement. The probed object was either targeted by none (motor-target mismatch), or one or more effectors (motor-target match).

We expect that bimanual hand movements, without an eye movement, to a common target will improve probe discrimination at the target location compared to unimanual hand movements. According to the independence of attention allocation by different effectors (Jonikaitis & Deubel, 2011; Bonfiglioli, Duncan, Rorden, & Kennett, 2002; Baldauf & Deubel, 2008b), we expect that a unimanual hand movement combined with an eye movement will similarly improve probe discrimination at the target location as compared to a bimanual pointing movement without an eye movement. Finally, when planning three effectors (eye movement plus bimanual movements), we expect probe discrimination to be enhanced even more than by the use of two effectors.

## Methods

### Participants

Twenty-nine human individuals participated in the experiment. We chose for a sample that was approximately three to six times bigger than in previous studies by Deubel and colleagues to ensure that a potential inconsistency in the results of the current article (see Khan et al., 2011) would not be due to power. All participants were Utrecht University students (age: *M* = 21.1, *SD* = 1.4; 23 females; 25 right-handed) and had normal or corrected-to-normal vision. Participants were naïve to the purpose of the experiment, gave written informed consent before participation, and received study credit after participation. The experiments conformed to the ethical principles of the Declaration of Helsinki and were approved by the local ethical committee of Utrecht University.

### Apparatus and material

**Figure 1.**
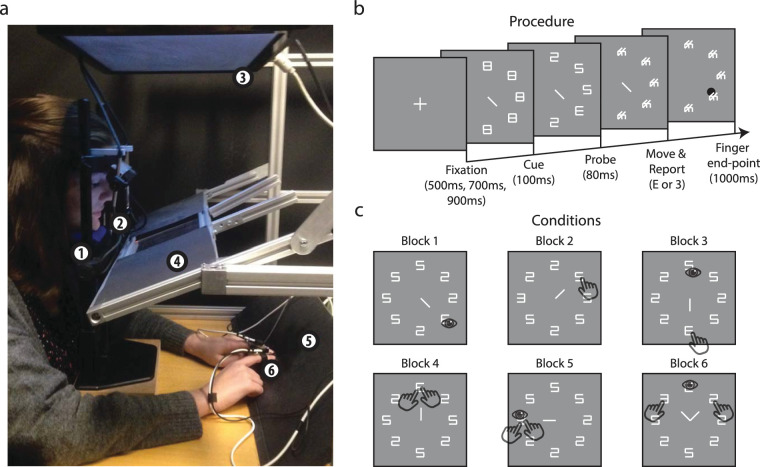
Experimental setup. (a) The experimental setup: A chin rest (1) ensured a fixed distance between the participant's head and screen. A head-mounted eye-tracker recorded gaze (2). Positioning the stimulus screen (3) above the half-transparent mirror (4) prevented disturbance of the tracking device's magnetic field and prevented the hands from blocking visual information. The mirror reflected the stimuli as if they appeared at the black surface (5) behind the mirror. A magnetic tracking device followed sensors attached to the participant's index fingers (6) to measure the endpoint of hand movements. (b) Procedure: The task of the participant was to fixate a cross and plan an eye or hand movement(s) to a location at which the probe (E or 3; motor-target match trial) or a distractor (5 or 2; motor-target mismatch trial) appeared. Movements were made during the mask period and finger endpoint feedback was provided afterward. (c) Examples of conditions per trial block: any possible combination between match (see block 1 example) versus mismatch conditions (see block 2 example), the number of effectors moving (one in blocks 1–2, two in blocks 3–4, and three in blocks 5–6), and the numbers of effectors to the probe's location (1, 0, 1, 2, 3, and 1, respectively in blocks 1–6 examples), produced a large variety of conditions. The displayed stimuli may mismatch in dimensions with the actual stimuli due to illustrative purposes.

The setup was comparable to the setup reported in Jonikaitis and Deubel (2011; see Figure 1a). Stimuli were generated on a 24-inch Iiyama ProLite E2482HS TFT screen (Tokyo, Japan) with a Dell OptiFlex 7040 computer (Round Rock, TX, USA) operating Windows 7 (Microsoft, Redmond, WA, USA) and Matlab version 2016a (Mathworks, Natick, MA, USA). The presentation monitor displayed 1920 × 1080 pixels at a 60-Hz refresh rate. Screen size was 52.3 cm in width and 29.4 cm in height (27.6 × 16.4 visual degrees), and the participant's viewing distance to the screen was fixed with a chin and forehead rest at 50 cm. Participants looked down at an angle of approximately 4° to a half-transparent mirror. The mirror reflected the screen's image displayed from above and blocked the sight of their own hands that were held behind the mirror. The 3D location of the tip of the participant's index fingers of both hands was measured with a miniBIRD motion tracker (Ascension Technologies Corp, St. Louis, MO, USA) at 100 Hz. Fingers could hit a black, wooden board that was positioned at 50 cm distance from the participant's eye to simulate the stimulus screen. An LED was attached to the tracker at the tip of the finger. The light was visible through the mirror. Gaze of the left eye was monitored with an EyeLink II head-mounted eye-tracker (SR Research, Osgoode, Ontario, Canada) at a rate of 250 Hz.

### Procedure, stimuli, and conditions

The goal of the experiment was to investigate whether bimanual pointing improves pre-movement probe recognition as compared to pointing with only one hand. We chose to follow procedures of a previous publication (Jonikaitis & Deubel, 2011) in which the probe is temporally masked and only presented for short durations (Figure 1b). Each trial started with the presentation of eight masks^1^ consisting of white lines showing an 8 (width: 0.9 visual degrees, height: 1.4°; luminance: 350 cd/m^2^) at an imaginary circle at 6.5° eccentricity from fixation, equally spaced from each other with an Euclidean distance of 5.1°. The fixation consisted of a white cross (350 cd/m^2^; 0.3° in diameter) and was presented on a gray background (175 cd/m^2^). After a fixation period with a duration randomly chosen from 500, 700, or 900 ms, the fixation was replaced by a 100 ms lasting cue(s) with the same size as the masks that indicated where the participant had to point and/or gaze. Participants were instructed to point and move gaze as accurate as possible. The cue period was followed by the presentation of a probe (3 or E) among distractors (2 or 5) for 80 ms and a mask period that lasted until the participant performed the saccade or pointing movement(s) and provided verbal feedback to the experimenter about the probe's identity. The experimenter recorded the response with a keyboard^2^. The LED(s) on the tip of the participant's finger(s) were then turned on for one second to provide feedback about the endpoint of their movements. Neither feedback was provided about saccadic endpoints, nor about the correctness of the identification of the probe. The next trial was initiated automatically after response.

Depending on the block of trials, either one effector (gaze in block 1; a single hand in block 2), two effectors (gaze and a single hand in block 3; two hands in parallel in block 4), or three effectors (gaze and both hands in block 5–6) could be moved congruently to the same location or incongruently to separate locations. As more effectors also meant more coordination and thus increased task difficulty, we ensured that the motor task would not become too difficult for participants by incrementally increasing the number of effector across blocks. To further control task difficulty, gaze always had to be allocated to a predefined location in the most difficult blocks 3–6, which was communicated to the participant before the start of each of these blocks. Blocks 1 and 2 consisted of 96 trials and blocks 3–6 of 192 trials. Because similar movements were performed in each block, participants could get acquainted with the motor task before increasing its difficulty in the next block. To prevent interference of a learning effect, we counterbalanced the order of blocks by decreasing the number of effectors in a second session that was performed by participants at least a day later but not later than a week. For the same reason we also counterbalanced the order of block 1 versus 2, 3 versus 4, and 5 versus 6 across participants. Participants practiced each block before starting the experiment. All blocks together summed up to a total of 1920 trials, performed in two separate sessions of approximately 1.5 hours each.

The probe appeared at the motor-target location in 50% of the trials in blocks 1 and 2, that is when only gaze or only a single hand was moved. In blocks 3 and 4, which involved two effectors moving, the probe's location matched at least one of the motor-targets locations in 66.7% of the trials (25% for gaze only and 25% for hand only, and 16.7% for combined eye/hand movements). In block 5, which involved three effectors moving to two possible targets (hand movements always coordinated to the same target), the probe's location matched at least one of the motor-targets locations in 66.7% of the trials (25% for gaze only, 25% for bimanual hands, and 16.7% for combined movements). In block 6, which involved three effectors moving, the probe's location matched at least one of the motor-targets locations in 66.7% of the trials (25% for gaze, 12.5% for left hand, 12.5% for right hand, 16.7% for combined movements). Only in blocks 4 and 6, in which the two hand effectors moved to independent locations, two cues were shown, one for each hand. In blocks 2 through 5 trials the cue indicated where the hands had to move and in block 1 where gaze needed to be allocated. Variations in cue, probe, and effector end-point locations produced a large variety of trial types. Figure 1c depicts several examples of trials per block (for an overview of all possible unique conditions, see Supplementary Figure S1). The block 1 example shows a motor-target match trial for a single effector (gaze), the block 2 example a mismatch trial for a single effector (hand), the block 3 example a match trial for one of the two effectors (gaze and hand), the block 4 example a match trial for two effectors (both hands; bimanual; note that in this example the cues for each hand overlap), the block 5 example a match trial for three effectors (gaze and both hands), and the block 6 example a cue-valid trial for one of three effectors.

### Analysis

The timing of hand movement onsets were detected by using a velocity threshold of 25 visual degrees per second (see Khan et al., 2011). Saccade onsets were detected with the default detection settings of the EyeLink 1000. We categorized the motor-target locations, probe locations, and effector type manipulations in three factors: (1) *motor-target match*, that is the probe's location matched (one) or mismatched (zero) a movement's end-point location; (2) *motor task* complexity, that is how many effectors (one, two, or three) moved towards a location(s); (3) *effector effectiveness*, that is how many of the effectors (zero, one, two, or three) were planned to move to the location where the probe appeared. We also added session number (1 or 2) as a fourth factor to take into account learning effects across sessions and we added participant number as a random effect. We used these five factors as independent variables for a generalized linear mixed effects regression model predicting whether a probe was correctly recognized or not as the dependent variable. Our main goal was to scrutinize to what degree the first three factors affected performance while keeping the model parsimonious. Note that we could have also chosen to leave out the factor *motor-target match* and regress the factor *effector effectiveness (0-3)* with a quadratic term. The quadratic function would then capture the larger difference in percent correct between zero and one effector as com the probe's location (Jonikaitis & Deubel, 2011). However, we chose to stick to a model with only linear parameters for the sake of simplicity. Also, different processes may be activated in motor-target mismatch trials than in motor-target match trials (e.g., a serial search through the fleeting image in working memory's visuospatial sketchpad), which can best be represented in the model as a separate, third factor (i.e., motor-target match probability). The predicted values for the Generalized Linear Mixed-Effects Model (GLME) consisted of a 1920 (trials) × 29 (number of participants) matrix with each value a correctly (1) or incorrectly (0) recognized probe. The GLME formula consisted of a logit function $\bar{y}=\frac{1}{1+e^{-y}}$ with $y=a_{intercept}+\beta_{match}*x_{match}+\beta_{motor}*x_{motor}+\beta_{eff}*x_{eff}+\beta_{session}*x_{session}+b_{participant}$. A two-way repeated measures analysis of variance was performed to examine the difference in the effects of hands versus gaze on performance (i.e., the factor *effector type*) in combination with either *motor task* or *effector efficiency*. Post-hoc statistical comparisons across conditions were performed with two-tailed, paired Student's *t*-tests (alpha = 0.05). Correlations between the average percent correct of ground truth and the modeled predictions across conditions were computed with Spearman's rho (which is here most appropriate due to the skewed distribution of data caused by the deviant percent correct for mismatch trials).

## Results

One participant scored at chance performance and was therefore removed from the analysis. All other participants correctly recognized the probe in 76% (*SD* = 6%) of all trials on average. Motor executions were valid (i.e., all end points were closer to the target than a distractor location, and movements had amplitudes of more than two visual degrees and onsets after probe offsets) in 58.3% (*SD* = 15.4%) of all trials on average. This percentage is comparable to previous studies (Khan et al., 2011; Elshout, Van der Stoep, Nijboer, & Van der Stigchel, 2020) and not surprising given the difficulty of the task. Nonetheless, we kept invalid trials for analyses for the sake of statistical power and because an inaccurate motor execution does not necessarily mean that the shift of attention was inaccurate. Including these excluded trials does not qualitatively alter the results.

The eyes and hands moved well after the disappearance of the probe with an average of 100 ms and 200 ms after probe offset, respectively (Saccade latency: 275 ± 55ms; reach latency: 372 ± 57 ms). Average saccade and reach amplitudes were 6.1 visual degrees (*SD* = 0.2°) and 5.8 visual degrees (*SD* = 0.3°), respectively. These numbers indicated that movement aim tended to undershoot with endpoints near the border rather than center (eccentricity = 6.5 visual degrees) of target locations.

**Figure 2.**
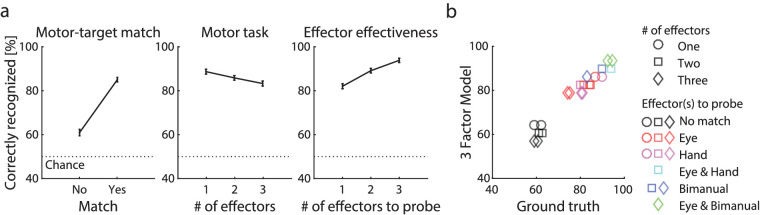
Results. (a) The average and standard error of percent correctly recognized probes as a function of each manipulated factor. The effect of motor-target match (left) suggests that allocating attention (and eventually an effector) to a probe location improves recognition performance. The detrimental effect of motor task (center) indicates that the more effectors moved, the more complex the motor task (i.e., the more difficult the task). Last, the positive effect of effector effectiveness indicates that the more effectors moved toward the probe's location, the better the performance. (b) These three factors (session here excluded) predicted the results very well.

**Figure 3.**
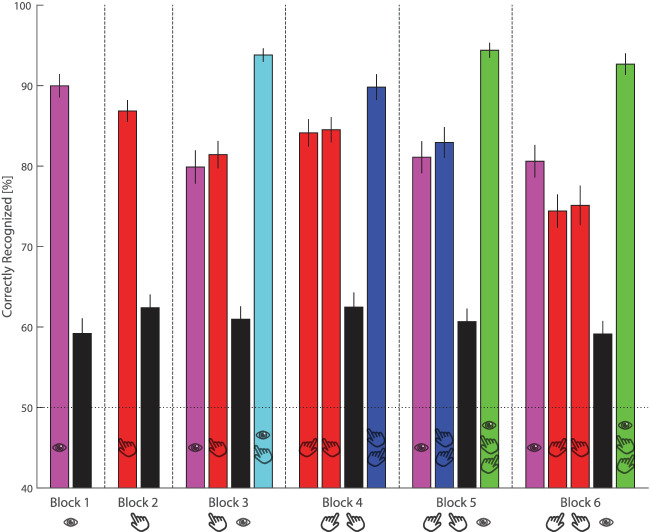
Results across blocks. Average and standard error of percent correctly recognized probes per condition and block. The icons below the x-axis indicate the moved effectors and the icons above the x-axis indicate which of these effectors were moved toward the probe's location. The horizontal dotted line indicates chance performance.

To double-check whether there was no effect of block order, we confirmed that participants that started with block 2 did not differ in average performance across blocks from participants that started with block 1 (*t*(27) = 1.31, *p* = 0.201). Note that performance was lower in session 1 as compared to 2, confirming a learning effect (*t*(28) = 5.36, *p* < 0.001). The generalized linear mixed effects model indicated significant effects of the intercept (α = 0.42, *p* < 0.001), and the factors motor-target match (*β*_match_ = 0.51, *p* < 0.001), motor task (*β*_motor_ = −0.16%, *p* < 0.001), effector effectiveness (*β*_eff_ = 0.59, *p* < 0.001), session number (*β*_session_ = 0.25, *p* < 0.001), and the participant-dependent intercept (*SD*_estimate_ = 0.35; *SD*_lower_= 0.27, *SD*_upper_ = 0.46). The average correctly recognized probes is plotted per factor in Figure 2a (except for session number), and all conditions significantly differed from each other per factor (for the most relevant post-hoc comparisons, see Supplementary Table S1; for a bar graph of the results per trial block, see Figure 3). On average performance increased by 24% (*SD* = 6%) if at least one effector was planned to move to a probe location and another 6% (*SD* = 3%) for each additional effective effector. Performance increased 4% (*SD* = 1%) from session 1 to session 2. Performance decreased 3% (*SD* = 3%) per effector moving (i.e., motor task difficulty), regardless of whether the effector targeted a probe location.

The correlation between the model's predictions and ground truth data points (averaged across participants; one data point per condition) are shown in Figure 2b. The nonparametric correlation coefficient was high and significant (*rho* = 0.95, *p* < 0.001). Next, we inspected the difference in probe recognition performance between gaze and hand effectors. For the condition that only gaze or only one hand was moved toward a probe (Supplementary Figure S2), we observed no significant main effect of *effector type* (i.e., gaze vs. hands) across the number of effectors (*F*(2,56) = 1.53, *p* = 0.227). However, a significant interaction between *effector type* and *motor task* (*F*(1,2,28) = 6.91, *p* = 0.002) indicated that gaze and hand had diverging effects on performance depending on how many effectors were moved in total. Post-hoc comparisons suggested that planning to allocate gaze to the probe improved performance 3% (*SD* = 8%) and 6% (*SD* = 14%) more than planning a single hand movement to the probe when in total one (*t*(28) = 2.01, *p* = 0.054) or three effectors (*t*(28) = 2.32, *p* = 0.028) were moved, respectively. Similarly, when gaze was one of two effectors that both targeted the probe, performance was better than when both effectors were hands (*t*(28) = 2.56, *p* = 0.016). Taken all results together, probe discrimination performance improved considerably when a motor-target location matched the probe's location and improved steadily by approximately seven percent per additional effector that targeted the probe's location. Adding gaze as an effector improved performance more than hands in most conditions. The more effectors were moved, whether or not towards the probe's location, the motor task also became more challenging, reducing the performance by approximately 4% per effector.

## Discussion

The main aim of the present study was to investigate whether the strength of the pre-motor shift of attention accumulates if an object is targeted by multiple effectors. Participants moved either gaze or a single hand (one effector), two hands or both gaze and a hand (two effectors), or gaze and two hands (three effectors) to objects in the periphery. We used performance on a discrimination task of a pre-saccadic probe on the objects as an index of the strength of the pre-saccadic shift of attention. Results showed that average discrimination performance decreased linearly per additional effector independent of motor-target match probabilities, but also increased linearly per additional effector that targeted the probed object. Here, we will discuss these findings in more detail and relate them to the individual aims of our study.

Our first aim was to replicate the independent allocation of attention by different effectors by showing separate attentional resources for eye and hand movements (Hanning et al., 2018; Jonikaitis & Deubel, 2011; Baldauf & Deubel, 2008a-2008b). We indeed observed that combining a unimanual movement with an eye movement improved probe discrimination at the target location compared to performance as a result of a unimanual movement or only an eye movement. The only result that we could not replicate is that the performance at saccadic target locations remained stable between the gaze-only condition in block 1, when only gaze was moved, and the same condition in block 3, when both gaze and one hand was moved. We observed a drop in performance in block 3, which we attribute to the increased motor task complexity as more effectors moved.

Secondly, we extended the idea of independent resources of the different effectors by adding a third effector to our experimental design. In our crucial condition, observers initiated three movements to the target location. Not only did they perform an eye movement in the direction of a fixed location, but they also executed a bimanual pointing movement (i.e., with their two hands) towards this common location. When performing such coordinated bimanual hand and eye movements, the presaccadic shift of attention was boosted even further compared to when two effectors are deployed. It is interesting to note that performance in the three effector conditions was significantly larger than the bimanual condition (compare green vs. dark blue bars in Figure 3), but not for the other two effector conditions that included an eye movement (compare green vs. cyan bars in Figure 3). This lack of an effect could potentially be explained by performance hitting ceiling level in the three effector conditions or because eye movements were easier than hand movements. The latter interpretation is consistent with earlier claims that gaze is more dominant in shifting attention (Khan et al., 2011). Alternatively, eye movements may have been easier than hand movements because gaze moved to a fixed location in most of the eye movement conditions. An additional explanation is that participants did not have to suppress the tendency to inspect targets during eye movement trials, a tendency that is difficult to suppress when fixation needs to be maintained in hand movement only trials.

Nonetheless, the overall results are completely in line with the conclusion that more movements to a probe location result in better probe discrimination performance. These findings provide additional evidence for the idea that both hands have independent resources to allocate attention (Baldauf & Deubel, 2008a; Baldauf & Deubel, 2008b; Jonikaitis & Deubel, 2011; Bonfiglioli et al., 2002). Possibly, recognition may improve even further when more effectors (e.g., feet, posture, or head movements) are moved to an object.

Our different conditions varied in their task difficulty as a function of the number of movements had to be coordinated in parallel. For instance, in our perhaps most difficult conditions, observers had to move their hands independently to different locations. As task difficulty increased as more effectors were moved, this clearly affected the performance: in the most complex and demanding blocks, for example when three effectors moved, the performance on trials with only one of the effectors targeting a probe was lower than in the blocks in which observers only had to execute one movement that targeted the probe. This effect was strongest for hand movements, while the detrimental effect of motor task complexity hit a floor for gaze movements (see Supplementary Figure S2). When only gaze targeted the probe in the three effectors condition, performance did not drop further as compared to the two effectors condition. This is likely due to the fact that gaze targeted one location throughout a block, which made it an easier motor task and thus more resilient to the increase in motor task complexity. Also note that the chance that an one effector targeted a probe (i.e., a motor-target match) varied across blocks (e.g., 50% only gaze or only hand in blocks 1–2; 25% for the one effector match conditions in all other blocks; 25% for the bimanual condition in block 5; a summed 25% for both individual hands in block 6 in blocks 3–4) and also varied across effectors in block 6 (25% gaze only; 12.5% either one of the hands). This might have affected performance: when one effector targets a probe more often, participants might start to rely more on it and therefore increase learning rates specifically for this effector. We denote this possibility as unlikely because we did not observe differences in performance between blocks with different motor-target match probabilities (e.g., gaze only in block 5 versus 6). Nonetheless, the motor-match probability is an interesting factor to manipulate and explore in future studies.

By including motor task complexity as a factor in our linear regression model, we could disentangle the effect of task difficulty from the effect of the congruence of the different effectors. This statistical approach clearly showed that, although a higher task difficulty led to a lower performance on the pre-saccadic identification of the probe, the number of effectors that moved towards the probe's location was positively associated with identification performance. Interestingly, task difficulty might actually be a crucial factor in determining whether the boost in attentional resources due to multiple effectors is observed. For instance, the design of the previous study that did not observe such an additional effect of multiple effectors (Khan et al., 2011) used a lower number of possible target locations which might have made the task less difficult compared to the current study and the study by Deubel and colleagues (Jonikaitis & Deubel, 2011; Hanning et al., 2018).

In the blocks in which relatively simple movements had to be performed, performance on the identification of the yet-to-appear object was not at ceiling level and comparable to (or even lower than) when performing complicated movements involving multiple effectors to the same target. There are two possibilities to explain these findings. First, it could be that the additional resources are simply not available when performing only one movement. Additional performance boosts can then only be released by the movement of more congruent effectors. Secondly, the boost is perhaps only present when the need arises. In the complex conditions when multiple effectors are involved, the increased task difficulty resulted in an impairment on the identification task which was mediated by an additional shift of premotor attention. Simply put, when the task is relatively easy, there is no need to allocate all available attentional resources to the target location, because performance is already relatively good (Baldauf & Deubel, 2008b). The full capacity of all resources is only required when task difficulty impairs performance.

Our results clearly show that performance of presaccadic perception at a specific location can be improved when coordinated congruent movements are made with multiple effectors. This may have interesting implications in daily life situations when attention is impaired (e.g., due to stroke or head trauma). For instance, patients with visual spatial neglect have difficulties allocating attention to their contralesional hemifield in the absence of sensory or motor defects (Heilman & Valenstein, 1979). Rehabilitation methods are originally focused on training eye movements to the affected hemifield (Kerkhoff & Schenk, 2012). Our data suggest that more attentional resources can be allocated if more effectors are directed to the affected hemifield than the eyes alone, which may improve perception.

To summarize, our results show that (1) the more complex the motor task, the worse the performance; (2) the more effectors planned to move to the yet-to-appear object, the more attentional resources it receives and the better it is perceived; and (3) the accumulation of benefits points at parallel and presumably independent mechanisms that evoke pre-motor shifts of attention.

## Supplementary Material

Supplement 1

Supplement 2

Supplement 3

## Footnotes

1 Because moving three effectors was a challenging task, we chose less possible probe locations than Jonikaitis & Deubel (2011) to ensure an acceptable level of task difficulty.

2 We thought that it would be too difficult for participants to also provide a nonverbal motor response to report the probe's identity (e.g., with their feet).
