# Supplementary material for: Two hands are better than one: Perceptual benefits by bimanual movements
Source: J Vis. 2020 Oct 15;20(10):16. doi: 10.1167/jov.20.10.16 (PMC7571320; doi:10.1167/jov.20.10.16)
Supplement: Supplement 1 [file jovi-20-10-16_s001.pdf]

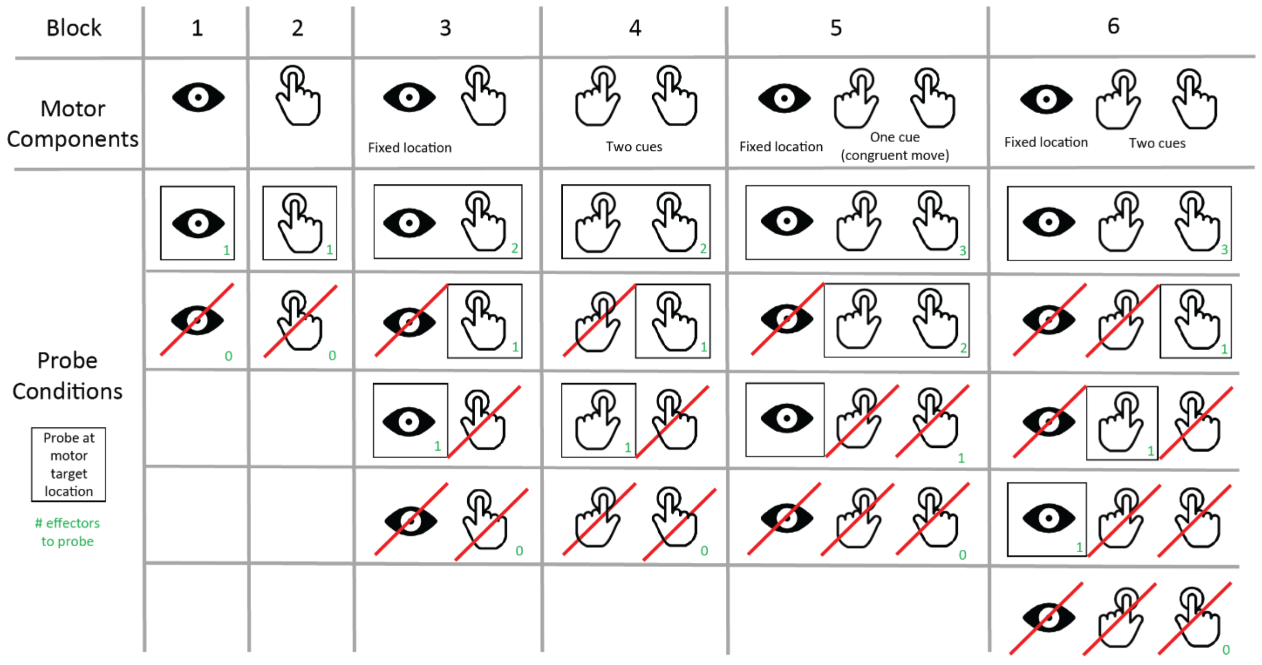

Figure S1. All cue, probe, and effector conditions across blocks. The icons in each table cell indicate which effectors were moved (one or two hands and/or an eye). The framed rectangles around the effectors indicate that this effector was moved to the probe, while the red-crossed effector(s) was moved to a different location than the probe. The green numbers at the bottom-right of each rectangle indicate the total number of effectors that moved to the probe.
