# Supplementary material for: Two hands are better than one: Perceptual benefits by bimanual movements
Source: J Vis. 2020 Oct 15;20(10):16. doi: 10.1167/jov.20.10.16 (PMC7571320; doi:10.1167/jov.20.10.16)
Supplement: Supplement 2 [file jovi-20-10-16_s002.pdf]

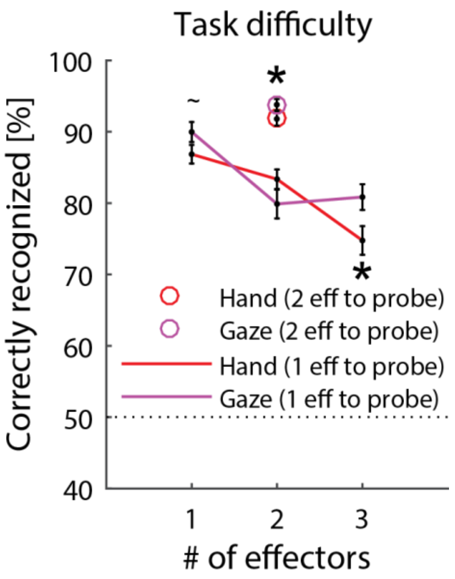

Figure S2. Gaze versus hand. The average and standard error of percent correctly recognized probes as a function of task difficulty, per condition that included at least a gaze movement (purple) or at least one hand movement (red), and per number of effectors to probe (one to probe: lines; two to probe: circles). Asterisks indicate the level of significance (~p = .05, *p < .05) of the difference between gaze (red) and hand (magenta).
