# Supplementary material for: Two hands are better than one: Perceptual benefits by bimanual movements
Source: J Vis. 2020 Oct 15;20(10):16. doi: 10.1167/jov.20.10.16 (PMC7571320; doi:10.1167/jov.20.10.16)
Supplement: Supplement 3 [file jovi-20-10-16_s003.pdf]

Table S1. Post-hoc t-test comparisons between conditions. Degrees of freedom were always 28. Sacc = saccade, L. = left, R. = right, Bi. = Bimanual or Saccade + hand, Tri. = all three effectors to probe. Note that only comparisons with right hands are shown when the effects of the left hand are comparable to the effects of the right hand. Significant comparisons are printed in bold.

**Conditions (block #)**		***t*-value**	***p*-value**
Uni. vs. Bi. block 3			
**Sacc (3)**	**Bi. (3)**	**7.55**	**< .001**
**R. Hand (3)**	**Bi. (3)**	**7.68**	**< .001**
Uni. vs. Tri.block 5			
**Sacc (5)**	**Tri. (5)**	**7.15**	**< .001**
**Bi. (5)**	**Tri. (5)**	**5.94**	**< .001**
Bi. vs. Bi. across blocks			
**Bi. (3)**	**Bi. (4)**	**2.56**	**.016**
**Bi. (3)**	**Bi. (5)**	**5.42**	**< .001**
**Bi. (4)**	**Bi. (5)**	**4.04**	**< .001**

**Conditions (block #)**		***t*-value**	***p*-value**
Uni. vs. Bi. Block 4			
**L. Hand (4)**	**Bi. (4)**	**4.29**	**< .001**
**R. Hand (4)**	**Bi. (4)**	**3.54**	**.001**
Uni. vs. Tri. block 6			
**Sacc (6)**	**Tri. (6)**	**5.71**	**< .001**
**R. Hand (6)**	**Tri. (6)**	**6.82**	**< .001**
Tri. vs. Tri. across blocks			
Tri. (5)	Tri. (6)	1.54	.134
Bi. vs. Tri. across blocks			
Bi. (3)	Tri. (5)	0.62	.542
Bi. (3)	Tri. (6)	0.96	.345
**Bi. (4)**	**Tri. (5)**	**3.55**	**.001**
**Bi. (4)**	**Tri. (6)**	**2.81**	**.037**
**Bi. (5)**	**Tri. (6)**	**4.83**	**< .001**
